# LC-MS/MS Profiles and In Vitro Biological Activities of Extracts of an Endemic Species from Turkey: *Stachys cretica* ssp. *anatolica*

**DOI:** 10.3390/plants10061054

**Published:** 2021-05-25

**Authors:** Ivana Carev, Cengiz Sarikurkcu

**Affiliations:** 1 Faculty of Chemistry and Technology, University of Split, Ruđera Boškovića 35, 21000 Split, Croatia; 2 NAOS Institute of Life Science, 355, rue Pierre-Simon Laplace, 13290 Aix-en-Provence, France; 3 Mediterranean Institute for Life Science, Meštrovićevo šetalište 45, 21000 Split, Croatia; 4 Faculty of Pharmacy, Afyonkarahisar Health Sciences University, Afyonkarahisar 03150, Turkey; sarikurkcu@gmail.com

**Keywords:** *Stachys*, antioxidant, phenolics, flavonoids, LC-MS/MS, tyrosinase, α-amylase

## Abstract

*Background*: Genus *Stachys* is one of the largest of the *Lamiaceae* family, having around 300 different plant species inhabiting areas with temperate and warm climates. The *Stachys* species in Turkey are represented with 81 taxa; 51 of them being endemic. Plants of the *Stachys* genus have been known for their biological activity and their use in ethnomedicine. *Methods*: The dominant components of *S. cretica* ssp. *anatolica* aqueous and methanol extracts were studied with the LC-MS/MS technique. *Results*: Chlorogenic acid, apigenin-7-glucoside and verbascoside present as the dominant polyphenols found in studied extracts. The prominent biological activity of the studied *S. cretica* ssp. *anatolica* methanol and aqueous extracts showed strong antioxidant activity and inhibition of enzymes tyrosinase and α-amylase, involved in skin disorders and diabetes mellitus type II. *Conclusions*: This study has proven that the aqueous and methanol extracts of *S. cretica* ssp. *anatolica* have prominent antioxidant activity, due to a high abundance of polyphenols. The strong antioxidant properties of *S. cretica* ssp. *anatolica* extracts show promising application for the pharmaceutical, food, and cosmetics industries.

## 1. Introduction

Plants are well-known in ethnopharmacology as source of biologically active compounds and present a valuable resource for many modern drugs derived from natural resources [1]. Plants, as is well-known, are influenced by a range of intrinsic and extrinsic factors, which, especially in officinal plants, can induce changes in the chemical composition and physiological activities of their essential oils [2]. The genus *Stachys* is one of the largest of the Lamiaceae family, having around 300 different plant species inhabiting areas with temperate and warm climates [3]. The *Stachys* species in Turkey is represented by 81 taxa; 51 of them being endemic [3].

In the Mediterranean, *Stachys* plant species are well-known in ethnomedicine, where they are used as food additives, herbal teas and medicine [4,5]. They have many different applications, ranging from use as disinfectants to wound healing, as astringents, anti-diarrheal, anti-spasmodic, to moderate earaches, in the inhibition of genital tumor development, cancerous ulcers, for mucus dissolving, cough relieving, alleviating the symptoms of asthma, curing fevers, in pain therapy, abdominal pain, dizziness, cramps, gout and menstrual disorders [4,5]. Due to the many biologically active chemical constituents *Stachys* species comprise, including phenolics, flavonoids, iridoids and diterpenoids, they can exhibit significant antifungal, antimicrobial, anti-inflammatory, antioxidant and cytotoxic activities [4,5].

It is well-known that phenolic compounds relate to the antioxidant activity of plant extracts. Therefore, they can be helpful in biological processes that include oxidation and oxidative stress, such as some diseases. The phenolic compounds are known to show potential effects against the development of cancer, cardiovascular diseases, skin problems, inflammations, bacterial infections and other health disorders, and can additionally influence enzymes tyrosinase and α-amylase [6,7,8,9,10,11,12]. 

Most of the research related to chemical compounds in *Stachys* species has dealt with volatile compounds. Sesquiterpene hydrocarbons, oxygenated monoterpenes and oxygenated sesquiterpenes were the main group of components found in several *Stachys* species: the presence of phenylethanoid glycosides, polyphenolics and flavonoids was reported, as well as common secondary metabolites. There are only few reports that list the phenolic components present in *Stachys* species, their antioxidant capacity and inhibition of enzymes. Thus far, phenolics have been studied in *Stachys cretica* L. ssp. *vacillans* Rech. Fil., *S. cretica* L. ssp. *smyrnaea* Rech. Fil., *S. cretica* ssp. *mersinaea* (Boiss.) Rech. Fil. [13,14,15].

Even though it is one of the largest Lamiaceae genus, research on the antioxidant potential of *Stachys* species has been very scarce to date [16]. The same is true for the inhibition of enzymes important in the deprivation of diabetes type 2 and hyperpigmentation. The discovery of new bioactive compounds can lead to new natural compounds as a resource for new drugs [1,9,17].

Regarding the phytochemical and ethnopharmacological value of *Stachys* species, this study analyzed methanol and aqueous extracts of *Stachys cretica* L. ssp. *anatolica* Rech. Fil. phenolic profile, total phenolics/flavonoids content, antioxidant activity and enzymes inhibitory potential of tyrosinase and α-amylase. To the best of our knowledge, this is the first such study from Turkey.

## 2. Results and Discussion

The phytochemical analysis of aqueous and methanol extracts from *S. cretica* ssp. *anatolica* have been screened for 31 standard compounds using coupled LC-MS/MS liquid chromatography, and the results are presented in Table 1.

**Figure 1:**
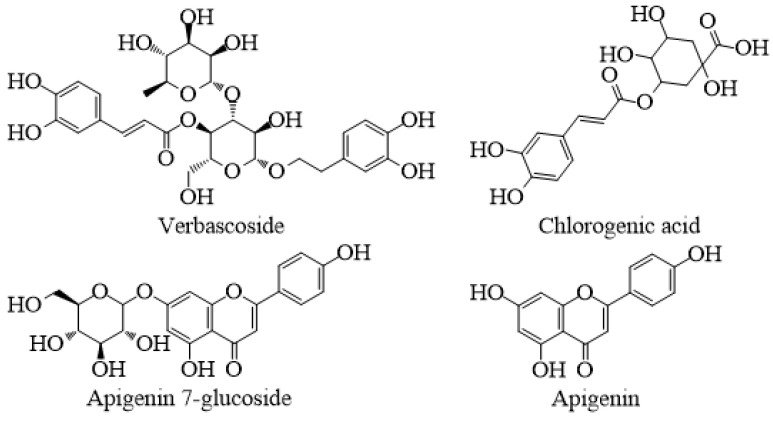
Major phenolic compounds identified in *S. cretica* ssp. *anatolica* extracts.

Previous studies showed that extraction with a solvent of different polarity resulted in a different yield of phenolic compounds [13,14,15,18,19,20]. The same conclusion was made in our study. The chemical composition of two samples extracted with the solvents of different polarities, water and methanol, showed significant differences in the yield of studied chemical compounds. In aqueous extract, 22 chemical compounds were detected, while in methanol extract, 25 were detected. The main constituents of both aqueous and methanol extracts were chlorogenic acid, apigenin-7-glucoside and verbascoside, presented in Figure 1.

The yield of all the main constituents in the studied extracts was significantly higher in methanol than in aqueous extract, showing 9437.77 µg/g of chlorogenic acid, 2313.87 µg/g of apigenin-7-glucoside and 25,402.03 µg/g of verbascoside. The difference in the chemical composition of the dominant components was seen in presence of caffeic acid and apigenin, which were present in high amounts in aqueous and methanol extracts, respectively. It is noteworthy to point out that the amount of syringic acid, luteolin-7 glucoside, pinoresinol and luteolin was also significantly higher in the methanol extract than in the aqueous extract.

The chemical composition of the plants can be helpful in their taxonomy, showing that the species and subspecies of the same taxa, genus and family have similar chemical compounds, which is also the case with *Stachys* species^4^. We will use this fact to compare our results with studied aqueous and methanol extracts from *S. cretica* ssp. *vacillans, S. cretica* ssp. *smyrnaea, S. cretica* ssp. *mersinaea, S. tmolea* (Boiss.), *S. byzantine* K. Koch, *S. iberica* Bieb. subsp. *iberica* var. *densipilosa* Bhattacharjee, *S. annua* L. spp. *annua* L. var. *annua* L., *S. balansae.* ssp. *balansae* (Boiss & Kotschy), *S. mardinensis* (Post) R.R.Mill., *S.* *megalodonta* Hausskn. & Bornm. ex P.H.Davis ssp. *mardinensis* Bhattacharjee and *S. thirkei* C. Koch [13,14,15,18,19,20,21,22].

Chlorogenic acid, the most abundant phenolic in the studied extracts, has been detected in all of the aforementioned and previously studied *Stachys* species, both methanol and aqueous extracts [13,15,18,19,20,21,22]. The range of the chlorogenic acid in mentioned methanol extracts was from 95.55 µg/g to 35,372 µg/g, while in aqueous extracts range from 426.30 µg/g to 17,772 µg/g. In all the previously studied *Stachys* species, chlorogenic acid was among the dominant components in both aqueous and methanol extracts, except in *S. cretica* ssp. *smyrnaea* methanol extract, where it showed the least abundance of all the studied *Stachys* species. Chlorogenic acid is the isomer of caffeoylquinic acid present in many different plants. It has been known to have neuroprotective, cardioprotective, hepatoprotective, anti-inflammatory, antibacterial, antiviral, antioxidant and other biological properties. Due to its proven biological activity, it can be used as a natural additive in the food and cosmetic industries, as well as for health benefits [23,24]. 

Apigenin-7-glucoside and verbascoside were previously reported only in *S. cretica* ssp. *vacillans* and *S. tmolea* methanol and aqueous extracts. In both mentioned species, these compounds were among the most dominant components. In other *Stachys* species, these compounds were not studied [13,18]. In *S. tmolea*, apigenin-7 glycoside showed the lowest yield of 34.15 µg/g in aqueous extract, while its yield of 2313.87 µg/g was highest in our study of *S. cretica* ssp. *anatolica* methanol extract. It is noteworthy to mention that the yield of 2116.10 µg/g in our studied *S. cretica* ssp. *anatolica* aqueous extracts was the highest recorded in the aqueous extracts examined so far. Verbascoside had the lowest yield in the aqueous extract of *S. tmolea* 1948.73 µg/g, while the highest yield of 47,289.47 µg/g was in the methanol extract of *S. cretica* ssp. *vacillans*. Verbascoside is caffeoyl phenylethanoid glycoside, and is very common for Lamiaceae plants. It has been known to have prominent biological activity which can be used in various industries as well as for health benefit [8]. 

Apigenin is commonly present in nature, very often as glycosylated flavonoid, and has been found in many plant tissues. Apigenin has been found in all of the hitherto studied *Stachys* species, in both methanol and aqueous extracts, showing higher yield in methanol extracts in general [13,15,18,19,20,21]. In the studied methanol extract of *S. cretica*, ssp. *anatolica* presents a dominant component with a yield of 2004.75 µg/g, while in aqueous extracts had significantly lower yield of 163.04 µg/g.

Caffeic acid is a very common phenolic compound well-known for its antioxidant properties. Caffeic acid, found in all of the previously studied *Stachys* species, has a significantly smaller yield than the dominant components of the studied extracts [13,18,19,20,21]. The studied *S. cretica* ssp. *anatolica* aqueous extract showed the highest yield of caffeic acid yield of 967.83 µg/g, among the Stachys species studied so far.

It is noteworthy to mention that methanol extract of the studied *S. cretica* ssp. *anatolica* had significantly higher yields of the 4-hydroxibenzoic acid, vanillic acid, syringic acid, vanillin, ferulic acid, luteoline-7-glucoside, hyperoside, pinoresinol and luteolin, in comparison to the aqueous extract. At the same time, the aqueous extracts had significantly higher yield of p-coumaric acid and rosmarinic acid compared to methanol extract.

The studied *Stachys* species showed significantly higher yields, and can be a good natural plant resource of the studied phenolics. All of the dominant components, found in studied extracts of *S. cretica* ssp. *anatolica*, have proven strong antioxidant activity. Their good antioxidant activity in the emulsion give them advanced arguments to be used in variety of biological and non-biological systems [8].

To connect the phytochemical composition of the studied *S. cretica* ssp. *anatolica* aqueous and methanol extracts with their biological activity, total phenol and total flavonoid content were determined using Folin–Ciocalteu and AlCl_3_ methods, respectively [25]. Antioxidant potential of both extracts was determined with six methods: ferrous ion chelating, CUPRAC, FRAP, DPPH, ABTS and phosphomolybdenum assays; results are presented in Table 2.

The extraction of phenols and flavonoids content depend on the solvent used. The studied aqueous extract contained the higher total amount of flavonoids and total amount of phenolic compounds, 30.20 mg quercetin equivalent; (QEs)/ g extract and 42.13 mg GAEs/g extract, respectively, than methanol extract, 24.62 mg REs/g extract and 27.13 mg GAEs/g extract, respectively, as shown in Table 3. 

It can be concluded that water seemed to be more suitable for extraction of total phenolics and flavonoids components in *S. cretica* ssp. *anatolica*. In some previous results, the methanol extracts were shown to be more effective as an extraction solvent for both phenolic and flavonoids, except with *S. byzantine* and *S. iberica* ssp. *iberica* var. *densipilosa* where aqueous extract yield more of the phenolics as in our study [13,15,18,19,20]. The amount of total flavonoids in our study is significantly higher than in previously studied S. cretica ssp. smyrnaea and S. tmolea, but roughly of the same range as in other previously studied Stachys species. The total phenolics were significantly higher than in in previously studied S. cretica ssp. smyrnaea, S. cretica ssp. vacillans and S. tmolea, but roughly of the same range or slighly lower than in other previously studied Stachys species [13,15,18,19,20].

The higher amount of phenolic and flavonoid compounds is directly correlated to a stronger antioxidant activity showed by aqueous extracts in all methods tested. This is in correlation with previous research where polyphenols have shown high antioxidant potential [8]. It is also interesting to note that the response of aqueous extract compared to methanol extract, in almost all the methods, was more or less doubled, as it is the ratio of the amount of phenolics in aqueous comparison to methanol extracts. In comparison with previously reported results on all of the studied antioxidative methods: CUPRAC, FRAP, DPPH, ABTS, phosphor-molybdenum and ferrous ion chelating, two of the studied extracts showed significantly higher antioxidative potential than most of the previously studied *Stachys* species [13,15,18,19,20]. An exception to this is *S. cretica* ssp. *vacillans*, where the results are similar and of the same range as in our study for all methods, but exactly the opposite for water and methanol extract, where methanol extract showed a higher antioxidative potential than the water extract [13].

Contrary to the antioxidant activity inhibition exhibited by both enzymes, tyrosinase and α-amylase was stronger with methanol extract (163.14 mg KAE/g extract and 5.12 mg ACE/g extract, respectively) than aqueous extract (306.05 mg KAE/g extract and 471.19 mg ACE/g extract, respectively) as shown in Table 3. The enzyme tyrosinase is the key enzyme in pigmentation and its inhibition could prevent the skin disorder hyperpigmentation. It is known that the flavonoids have prominent role as natural inhibitors of this enzyme, therefore herbal extracts could be natural resource for treatment of this skin disorder [9,26]. The enzyme α-amylase is important in glycemic control in diabetes mellitus type II. It is also known that digesting the polyphenols can be helpful in preventing and treating diabetes mellitus type II [7,11]. In our study, we have proven that the aqueous and methanol extracts of *S. cretica* ssp. *anatolica* have polyphenols that can be used for the inhibition of both studied enzymes. 

## 3. Materials and Methods

### 3.1. Plant Material

Aerial parts of *Stachys cretica* L. ssp. *anatolica* Rech. Fil. (Lamiaceae) were collected from Burdur-Turkey in 02 June 2015 (862 m, 37°39′05″ N 30°03′37″ E). This coordinated system belongs to WGS84 geographic system. The species were identified by Dr. Olcay Ceylan and stored at the Herbarium of Mugla Sitki Kocman University (Turkey) under the accession no. O.5306. The aerial parts of *S. cretica* ssp. *anatolica* were air-dried in the shade until stable weight, then cut into small pieces with a laboratory mill before solvent extraction. 

### 3.2. Solvent Extraction

The methanol extraction of the aerial parts of *S. cretica* ssp. *anatolica* was done by maceration for 24 h and then condensed using reduced pressure. The water extract was prepared by infusion in boiling deionized water for 15 min and the remaining solution was filtered then lyophilized as previously described [27]. Five grams of the aerial parts were mixed with 100 mL of solvent (1:20) and agitation was set to 150 rpm. The obtained extracts were stored at +4 °C for further analyses.

### 3.3. Total Flavonoid and Phenolic Contents

Using previously reported experimental conditions, an aluminum chloride method was used for total flavonoid content (TFC) while total phenolic content (TPC) was determined with Folin–Ciocalteu reagent (FCR) (Sigma-Aldrich, Germany) reagent and the results were expressed as equivalents of quercetin (Sigma-Aldrich, Steinheim am Albuch, Germany) and gallic acid (Sigma-Aldrich, Steinheim am Albuch, Germany), respectively, as previously described [25].

### 3.4. Liquid Chromatography–Electrospray Tandem Mass Spectrometry (LC–ESI–MS/MS) Analysis

Analysis of the selected phytochemicals in the extracts was carried out by an Agilent Technologies 1260 Infinity liquid chromatography system (Agilent Technologies, Inc., Frederick, USA) hyphenated to a 6420 Triple Quad mass spectrometer (Thermo Fisher Scientific, Lafayette, USA) on which a chromatographic separation on a Poroshell 120 EC-C18 (100 mm × 4.6 mm I.D., 2.7 μm) column (Agilent Technologies, Inc., Frederick, USA) was performed by using the analytical conditions reported previously [28,29].

### 3.5. Enzyme Inhibition Activity

For enzyme inhibitory activities, the extracts were analyzed toward α-amylase and tyrosinase (Sigma-Aldrich, Steinheim am Albuch, Germany) based on previous experimental conditions [30,31]. The enzyme inhibitory activity is expressed as mg standard equivalent/g extract. Kojic acid and acarbose (Sigma-Aldrich, Steinheim am Albuch, Germany) were used as positive controls.

### 3.6. Antioxidant Activity

The antioxidant properties were determined using the following assays: Total antioxidant capacity by phosphor-molybdenum method, cupric ion (CUPRAC) and ferric ion reducing power (FRAP), scavenging ability toward 2,2 Diphenyl 1 picrylhydrazyl (DPPH) radical; [2,2′-Azino-bis(3-ethylbenzthiazoline-6-sulfonic acid)] (ABTS.^+^) free radical scavenging and ferrous ion chelating [32,33,34,35,36,37,38]. The antioxidant activity is expressed as mg standard equivalent/g extract. Trolox and ethylenediaminetetraacetic acid (disodium salt) (EDTA) were used as positive controls. Reagents of antioxidant activity tests were purchased from Sigma-Aldrich (Germany).

### 3.7. Statistical Analysis

Statistical analysis was performed using SPSS software v22.0 for Windows (IBM Corp., Armonk, NY, USA). All results were expressed as mean ± standard deviation. Statistical significance was tested by Student’s t-test. Values were considered significant when *p*-value is lower than 0.05.

## 4. Conclusions

Plants of the *Stachys* genus are known for their biological activity, due to which there is a long tradition of using these species in ethnomedicine. This study has proven that the aqueous and methanol extracts of *S. cretica* ssp. *anatolica* have prominent antioxidant activity due to a high abundance of polyphenols. The dominant components of *S. cretica* ssp. *anatolica* aqueous and methanol extracts, studied with LC-MS/MS technique were: chlorogenic acid, apigenin-7-glucoside and verbascoside. The yield of dominant components, and most of the studies of polyphenols in the studied extracts, was shown to be higher in methanol than in aqueous extract, while total phenolic content, total flavonoids content and response to all the studied antioxidant methods, was higher in the aqueous extract. This is the case because we have chosen to quantify only certain chemical compounds found in these extracts. Future studies should clear out other phytochemicals present in the extracts, and their contribution to the prominent biological activity of the studied *S. cretica* ssp. *anatolica* methanol and aqueous extracts. The antioxidant properties of these extracts can be used for human health benefits as well as in various different industries. Extracts can also be used as mixtures or as a natural resource of polyphenols that can inhibit enzymes tyrosinase and α-amylase to prevent skin disorders as well as diabetes mellitus type II. From the presented results, it is evident that *S. cretica* ssp. *anatolica* is promising source of the chemical compounds whose application can be found in the pharmaceutical, food, and cosmetics industries. 

## Figures and Tables

**Table 1:** Concentration (µg/g extract) of selected phytochemicals in *S. cretica* ssp. *anatolica* extracts ^x.^.

Compound	Water	Methanol
Gallic acid	5.60 ± 0.10 ^b^	33.06 ± 0.93 ^a^
Protocatechuic acid	363.46 ± 0.09 ^a^	193.92 ± 1.31 ^b^
3,4-Dihydroxyphenylacetic acid	18.57 ± 0.07 ^a^	6.70 ± 0.24 ^b^
(+)-Catechin	nd	nd
Pyrocatechol	nd	nd
Chlorogenic acid	7252.19 ± 466.89 ^b^	9437.77 ± 407.11 ^a^
2,5-Dihydroxybenzoic acid	9.04 ± 0.49 ^a^	8.38 ± 0.52 ^a^
4-Hydroxybenzoic acid	43.37 ± 0.06 ^b^	97.26 ± 1.07 ^a^
(-)-Epicatechin	nd	nd
Caffeic acid	967.83 ± 10.86 ^a^	124.56 ± 0.23 ^b^
Vanillic acid	nd	857.97 ± 31.36
Syringic acid	91.45 ± 5.44 ^b^	512.43 ± 9.85 ^a^
3-Hydroxybenzoic acid	4.86 ± 0.17 ^b^	10.11 ± 0.14 ^a^
Vanillin	nd	81.02 ± 2.15
Verbascoside	15447.91 ± 102.41 ^b^	25402.03 ± 163.12 ^a^
Taxifolin	nd	nd
Sinapic acid	2.93 ± 0.47 ^b^	5.71 ± 0.01 ^a^
*p*-Coumaric acid	177.34 ± 2.32 ^a^	22.21 ± 0.59 ^b^
Ferulic acid	461.08 ± 5.84 ^b^	665.44 ± 7.72 ^a^
Luteolin 7-glucoside	67.08 ± 1.95 ^b^	225.19 ± 3.39 ^a^
Hesperidin	nd	nd
Hyperoside	101.51 ± 0.72 ^b^	252.49 ± 2.74 ^a^
Rosmarinic acid	68.22 ± 0.97 ^a^	6.58 ± 1.72 ^b^
Apigenin 7-glucoside	2116.10 ± 16.02 ^b^	2313.87 ± 0.48 ^a^
2-Hydroxycinnamic acid	nd	nd
Pinoresinol	12.58 ± 0.89 ^b^	204.85 ± 16.38 ^a^
Eriodictyol	0.69 ± 0.01 ^b^	3.52 ± 0.15 ^a^
Quercetin	5.22 ± 0.40 ^a^	8.73 ± 1.22 ^a^
Luteolin	62.71 ± 3.01 ^b^	859.93 ± 31.06 ^a^
Kaempferol	nd	4.65 ± 0.71
Apigenin	163.04 ± 5.53 ^b^	2004.75 ± 59.35 ^a^

^x^ The mean values followed by the same superscripts within a row do not differ, according to the Student’s *t*-test at 5% significance level. nd; not detected.

**Table 2:** Antioxidant activities of *S. cretica* ssp. *anatolica* extracts ^x.^.

Assays	Water	Methanol
Ferrous ion chelating (mg EDTAE/g extract)	68.38 ± 0.69 ^a^	53.64 ± 1.35 ^b^
CUPRAC reducing power (mg TE/g extract)	532.56 ± 5.83 ^a^	246.13 ± 2.80 ^b^
FRAP reducing power (mg TE/g extract)	303.28 ± 1.48 ^a^	127.20 ± 7.18 ^b^
DPPH radical scavenging (mg TE/g extract)	249.89 ± 1.52 ^a^	80.44 ± 3.04 ^b^
ABTS radical scavenging (mg TE/g extract)	276.68 ± 12.35 ^a^	112.19 ± 4.31 ^b^
Phosphomolybdenum (mg TE/g extract)	801.69 ± 31.26 ^a^	546.31 ± 13.99 ^b^

^x^ The mean values followed by the same superscripts within a row do not differ, according to the Student’s *t*-test at 5% significance level. TE and EDTAE; Trolox and Ethylenediaminetetraacetic acid (disodium salt) equivalents, respectively.

**Table 3:** Extraction yield, enzyme inhibition activity, total phenolic and flavonoid contents of *S. cretica* ssp. anatolica extracts ^x.^.

Assays	Water	Methanol
Yield (%)	12.25	5.45
Total flavonoids (mg QE/g extract)	30.20 ± 0.01 ^a^	24.62 ± 0.05 ^b^
Total phenolics (mg GAE/g extract)	42.13 ± 2.04 ^a^	27.13 ± 0.68 ^b^
Tyrosinase inhibition (mg KAE/g extract)	163.14 ± 10.55 ^b^	306.05 ± 4.94 ^a^
α-Amylase inhibition (mg ACE/g extract)	5.12 ± 0.59 ^b^	471.19 ± 28.62 ^a^

^x^ The mean values followed by the same superscripts within a row do not differ, according to the Student’s *t*-test at 5% significance level. GAE, QE, KAE, and ACE; gallic acid, quercetin, kojic acid, and acarbose equivalents, respectively.

## Data Availability

No new data were created or analyzed in this study. Data sharing is not applicable to this article.
